# Effectiveness of multi-drug regimen chemotherapy treatment in osteosarcoma patients: a network meta-analysis of randomized controlled trials

**DOI:** 10.1186/s13018-017-0544-9

**Published:** 2017-03-29

**Authors:** Xiaojie Wang, Hong Zheng, Tao Shou, Chunming Tang, Kun Miao, Ping Wang

**Affiliations:** 1 Department of Medical Oncology, the First People’s Hospital of Yunnan Province, Affiliated Hospital of Kunming University of Science and Technology, Kunming, 650032 China; 2 Department of Thoracic Surgery, the First People’s Hospital of Yunnan Province, Affiliated Hospital of Kunming University of Science and Technology, No.157 Jinbi Road, Kunming City, 650032 Yunnan Province China

**Keywords:** Osteosarcoma, Chemotherapy drugs, Progression-free survival, Overall survival, Meta-analysis

## Abstract

**Background:**

Osteosarcoma is the most common malignant bone tumour. Due to the high metastasis rate and drug resistance of this disease, multi-drug regimens are necessary to control tumour cells at various stages of the cell cycle, eliminate local or distant micrometastases, and reduce the emergence of drug-resistant cells. Many adjuvant chemotherapy protocols have shown different efficacies and controversial results. Therefore, we classified the types of drugs used for adjuvant chemotherapy and evaluated the differences between single- and multi-drug chemotherapy regimens using network meta-analysis.

**Methods:**

We searched electronic databases, including PubMed (MEDLINE), EmBase, and the Cochrane Library, through November 2016 using the keywords “osteosarcoma”, “osteogenic sarcoma”, “chemotherapy”, and “random*” without language restrictions. The major outcome in the present analysis was progression-free survival (PFS), and the secondary outcome was overall survival (OS). We used a random effect network meta-analysis for mixed multiple treatment comparisons.

**Results:**

We included 23 articles assessing a total of 5742 patients in the present systematic review. The analysis of PFS indicated that the T12 protocol (including adriamycin, bleomycin, cyclophosphamide, dactinomycin, methotrexate, cisplatin) plays a more critical role in osteosarcoma treatment (surface under the cumulative ranking (SUCRA) probability 76.9%), with a better effect on prolonging the PFS of patients when combined with ifosfamide (94.1%) or vincristine (81.9%). For the analysis of OS, we separated the regimens to two groups, reflecting the disconnection. The T12 protocol plus vincristine (94.7%) or the removal of cisplatinum (89.4%) is most likely the best regimen.

**Conclusions:**

We concluded that multi-drug regimens have a better effect on prolonging the PFS and OS of osteosarcoma patients, and the T12 protocol has a better effect on prolonging the PFS of osteosarcoma patients, particularly in combination with ifosfamide or vincristine. The OS analysis showed that the T12 protocol plus vincristine or the T12 protocol with the removal of cisplatinum might be a better regimen for improving the OS of patients. However, well-designed randomized controlled trials of chemotherapeutic protocols are still necessary.

**Electronic supplementary material:**

The online version of this article (doi:10.1186/s13018-017-0544-9) contains supplementary material, which is available to authorized users.

## Background

Osteosarcoma is the most common type of primary malignant bone tumour. It exhibits a high metastasis rate and is frequently detected in adolescents at sites of rapid bone growth [1, 2]. Although osteosarcoma is frequently treated by surgical joint amputation or disconnection, the prognosis remains poor in patients with metastatic osteosarcoma [3]. Therefore, the ultimate treatment of this disease not only depends on primary tumour control but also the removal of small metastases. Thus, adjuvant chemotherapy combined with the surgical removal of the primary tumour is needed to reduce the size of the tumour, clear the metastases, and improve progression-free survival (PFS) and overall survival (OS).

Osteosarcoma is also a relatively drug-resistant tumour, and the treatment effect of single-drug chemotherapy is not ideal [4, 5]. Thus, multi-drug regimens are necessary to control tumour cells at various stages of the cell cycle, eliminate local or distant micrometastases, and reduce the emergence of drug-resistant cells [6]. Several systematic reviews have examined osteosarcoma chemotherapy, but the results are controversial. A previous study suggested that ifosfamide-based chemotherapy could significantly improve the PFS and OS of osteosarcoma patients [7]. However, recent traditional meta-analyses have not determined whether ifosfamide application and chemotherapy have similar histological response rates and 5-year PFS and OS in non-metastatic and primary osteosarcoma patients; thus, ifosfamide is not recommended [8–10]. Additionally, in a systematic review concerning the dose of chemotherapy drugs, high-dose drugs did not significantly improve the PFS and OS of patients compared to moderate-dose drugs [11–13]. Thus, additional studies are needed to resolve these controversies.

From the 1970s to the present, many adjuvant chemotherapy protocols have shown various efficacy differences and controversial results. No definitive evidence exists regarding which treatment is more advantageous for clinical application [14, 15]. The aim of the present study was to analyse the existing chemotherapy protocol through direct and indirect comparisons to guide clinical application. However, an analysis of each type of chemotherapy protocol is too complex and cumbersome. Therefore, in the present study, we classified the types of drugs used in adjuvant chemotherapy and evaluated the differences between single or multi-drug chemotherapy regimens using a network meta-analysis.

## Methods

This network meta-analysis was performed in accordance with Preferred Reporting Items for Systematic Reviews (PRISMA) statement [16].

### Data search strategy and selection criteria

Two authors independently performed the literature search through November 2016 using electronic databases, including PubMed (MEDLINE), EmBase, and the Cochrane Library, with the keywords “osteosarcoma”, “osteogenic sarcoma”, “chemotherapy”, and “random*”, without language restriction. The bibliographies of the obtained publications and relevant reviews were also assessed to ensure that no relevant studies were inadvertently omitted. The publications included in the present study met the following criteria: (1) randomized controlled trial (RCT) design; (2) inclusion of osteosarcoma patients; (3) examination of two or more groups using different single- or multi-drug regimens; and (4) inclusion of PFS or OS as an outcome. The exclusion criteria consisted of the following: (1) non-RCT studies; (2) studies including patients with other types of sarcomas, such as Ewing sarcoma; (3) non-chemotherapy controlled studies, such as surgery or radiotherapy controlled studies; (4) studies comparing the same chemotherapeutic drug type, such as a drug dose-related study; and (5) non-desired outcome studies. Additionally, reviews, comments, case reports, basic studies, and conference reports were also excluded.

### Data extraction

Two authors independently extracted the following information from eligible studies: first author’s name, publication year, location, research time, study register or abbreviation, sample size, average age, ratio of males, type of disease, experimental intervention, control, and follow-up. In the present analysis, the major outcome was PFS, and the secondary outcome was OS, as some patients changed the initial randomized treatment after disease progression. We assessed the methodological quality of the included trials using the Cochrane Collaboration’s tool, which assigns grades of “high risk”, “unclear risk”, or “unclear risk” of bias across the seven specified domains [17].

### Statistics analysis

We initially conducted a pairwise meta-analysis using a random effect model, as this model is likely the most appropriate and conservative methodology accounting for between-trial heterogeneity within each comparison [18]. For dichotomous outcomes, odds ratios (ORs) or logarithm transformation with 95% confidence intervals (CIs) were calculated to determine the sizes of the effects. We also used a random effect network meta-analysis for mixed multiple treatment comparisons because this analysis fully preserves the within-trial randomized treatment comparisons in each trial [19]. To rank the treatments for each outcome, we used the surface under the cumulative ranking (SUCRA) probabilities [20]. Comparison-adjusted funnel plots were used to determine whether small-study effects were present in the analysis conducted in the present study [21]. All tests were two-tailed, and a *p* value of less than 0.05 was considered statistically significant. Data analyses were performed using STATA software (version 14.0; Stata Corporation, College Station, TX, USA).

## Results

### Literature search

**Fig. 1:**
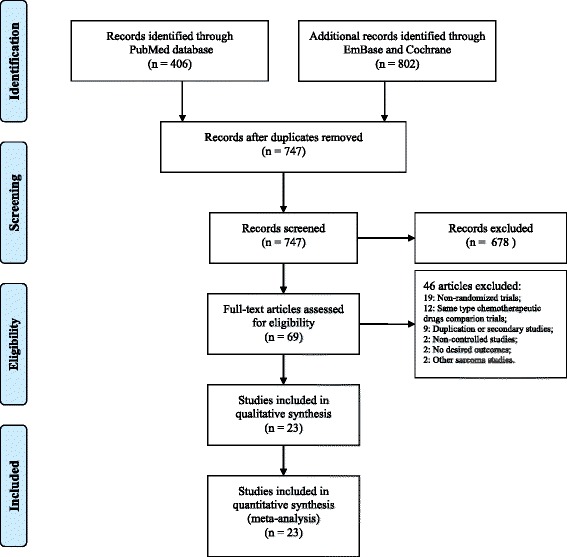
PRISMA flowchart illustrating the selection of studies included in the present analysis

In the present study, 747 articles were identified after the duplicates were removed. A total of 678 articles were excluded after the titles and abstracts were screened. The full texts of the remaining 69 articles were assessed, and the following types of studies were removed: non-randomized design (19); comparisons of the same type of chemotherapeutic drug (12); duplications or secondary studies (9); non-controlled studies (2); no desired outcomes (2); and other sarcoma studies (2). Eventually, 23 articles assessing a total of 5742 patients were included in the present systematic review [22–44] (Fig. 1, Table 1).

**Table 1:** Characteristics of subjects in eligible studies

Author	Year	Location	Research time	Study register/abbreviation	Sample size	Average age^a^	Male/Female	Type of disease	Follow-up
Yan Zhang [22]	2013	China	2007–2008	NA	76	24.4 ± 1.7	44/32	Enneking II-III	5 years
Neyssa M. Marina [23]	2016	International	2005–2011	EURAMOS-1	2260 (618)	4–40	365/253	High grade	62–63 months
Sophie Piperno-Neumann [24]	2016	France	2007–2014	OS2006	318	15.4 (5.8–50.9)	179/136	High grade	3.9 years
Stefan S. Bielack [25]	2015	International	2005–2011	EURAMOS-1	2260 (1041)	14 (11–16)	421/295	High grade	44 months
Alessandra Longhi [26]	2014	Italy	2007–2011	EudraCT:2006-002676-18	20	34 (11–65)	11//9	Postrelapse	73 months
J.S. Whelan [27]	2012	Europe	1982–2002	EOI (BO02/80831)	179	3–40	102/77	High grade	9.4 years
				EOI (BO03/80861)	391	3–38	261/130	High grade	9.4 years
Hui Zhao [28]	2010	China	2002–2007	NA	32	18.5 (7–68)	16/16	Lung metastasis	60 months
Alexander J. Chou [29]	2009	USA	2001–2005	CCG/POG (INT-0133)	91	<30	56/35	High-grade intramedullary metastasis	89 months
Paul A. Meyers [30]	2008	USA	2001–2005	CCG/POG (INT-0133)	662	13 (1–30)	361/301	High grade, Non-metastasis	7.7 years
Marie-Cecile Le Deley [31]	2007	France	1994–2001	SFOP-OS94 (NCT00180908)	234	13.2 (3.1–19.5)	131/103	High grade	77 months
Paul A. Meyers [32]	1998	USA	1986–1993	MSKCC (T12) protocol	73	15.8 (4.6–36.4)	42/31	High grade	91.4 months
Robert L. Souhami [33]	1997	International	1986–1991	EOI (T10) protocol	407	NA	261/130	High grade, Non-metastasis	5.6 years
Michael P. Link [34]	1993	International	1982–1984	MIOS	36	NA	NA	High grade, Non-metastasis	4–8 years
John H. Edmonson [35]	1984	USA	1976–1980	Mayo Clinic	38	17 (9–62)	24/14	Postoperation	31–74 months
K. Winkler [36]	1984	Germany	1979–1982	COSS-80	116	14 (5–24)	69/47	High grade	30 months
F. Eilber [37]	1987	USA	1981–1984	NA	112	15 (4–75)	44/15	Non-metastasis	2 years
D.R. Sweetnam [38]	1986	UK	1975–1981	NA	194	1–40	111/83	Lung metastasis	26–94 months
K. Winkler [39]	1988	Germany	1982–1984	COSS-82	125	14	73/52	Osteosarcoma	6 years
Vivien H.C. Bramwell [40]	1992	Canada	1983–1986	EOI	198	NA	114/84	High grade	5 years
John C. Ivins [41]	1976	USA	1974–1975	Mayo Clinic	26	NA	NA	Osteosarcoma	15 months
C. Jasmin [42]	1978	France	1976-	EORTC	27	18 (9–28)	13/14	Osteosarcoma	2 years
Gilchrist GS [43]	1978	USA	NA	NA	32	NA	NA	Osteosarcoma	753 days
J.M.V. Burgers [44]	1988	Netherlands	1978–1983	EORTC-SIOP03 (20781)	140	1–30	87/53	Osteosarcoma	5 years

*Abbreviations*: *CCG* Children’s Cancer Group, *COSS* Cooperative Osteosarcoma Study Group, *EOI* the European Osteosarcoma Intergroup, *EORTC* European Organization for Research on Treatment of Cancer, *EURAMOS-1* The European and American Osteosarcoma Study Group, *MIOS* the Multi-institutional Osteosarcoma Study, *MSKCC* Memorial Sloan-Kettering Cancer Center, *SFOP* Societe Francaise d’Oncologie Pediatrique, *SSG* the Scandinavian Sarcoma Group, *NA* not available

^a^Mean ± standardization; median (minimum-maximum); minimum-maximum

The included studies were published from 1976 to 2016 and were researched from 1974 to 2014. The analysis contained several multicentre large-scale studies, such as The European and American Osteosarcoma Study Group-1 (EURAMOS-1), Osteosarcoma 2006 (OS2006), and the Symposium of the Cooperative Osteosarcoma Study Group (COSS-80). Many studies contained duplicate reports. Thus, we included relatively recently published studies and referred to the outcomes of the duplicate reports. All age groups of patients were included, and slightly more men than women were included. All studies included patients with osteosarcoma defined according to a pathological diagnosis. In addition, four studies included osteosarcoma patients without metastasis, two studies included metastasis patients, and one study included relapse patients. Most studies initiated chemotherapy prior to surgery. The longest median follow-up period was 9.4 years (Table 1). All included studies had an RCT design without blinding, and most randomizations were not rigorous. However, the assessed outcome was objective; thus, the overall quality of the included studies was not ideal but was acceptable (Additional file 1: Figure S1).

For chemotherapeutic drug application, we investigated all types of drugs used in the intervention arms and classified each of the drugs of the experimental arms by alphabetical order. The present study did not include a comprehensive analysis, reflecting the characteristics of applied chemotherapeutic protocols, as most application stages, durations, and dosages of drugs were different in different protocols (Table 2). Drugs showing no chemotherapeutic effect, such as granulocyte colony-stimulating factor (G-CSF) and muramyl tripeptide, were excluded. Drugs that may be included in chemotherapy, such as mistletoe, were included in the present analysis.

**Table 2:** Interventions and abbreviations for eligible studies

Author	Year	Study register/short name	Intervention	Abbr.	Control	Abbr.
Yan Zhang [22]	2013	NA	Adriamycin; cisplatin; ifosfamide; recombinant human endostatin	APIR	Adriamycin; cisplatin; ifosfamide	API
Neyssa M. Marina [23]	2016	EURAMOS-1	Adriamycin; methotrexate; cisplatin	AMP	Adriamycin; methotrexate; cisplatin; ifosfamide; etoposide	AMPIE
Sophie Piperno-Neumann [24]	2016	OS2006	Methotrexate; ifosfamide; etoposide; zoledronate	MIEZ	Methotrexate; ifosfamide; etoposide	MIE
			Adriamycin; cisplatin; ifosfamide; zoledronate	APIZ	Adriamycin; cisplatin; ifosfamide	API
Stefan S. Bielack [25]	2015	EURAMOS-1	Adriamycin; methotrexate; cisplatin; interferonα-2β	AMPF	Adriamycin; methotrexate; cisplatin	AMP
Alessandra Longhi [26]	2014	EudraCT:2006-002676-18	*Viscum album*	V	Etoposide	E
J.S. Whelan [27]	2012	EOI (BO02/80831)	Adriamycin; methotrexate; cisplatin	AMP	Adriamycin; cisplatin	AP
		EOI (BO03/80861)	Adriamycin; bleomycin; cyclophosphamide; dactinomycin; methotrexate; cisplatin; vincristine	ABCDMPL	Adriamycin; cisplatin	AP
Hui Zhao [28]	2010	NA	Cisplatin; pirarubicin	PT	Ifosfamide; pirarubicin	IT
Alexander J. Chou [29]	2009	CCG/POG (INT-0133)	Adriamycin; methotrexate; cisplatin	AMP	Adriamycin; cisplatin; methotrexate; ifosfamide	AMPI
Paul A. Meyers [30]	2008	CCG/POG (INT-0133)	Adriamycin; methotrexate; cisplatin	AMP	Adriamycin; cisplatin; methotrexate; ifosfamide	AMPI
Marie-Cecile Le Deley [31]	2007	SFOP-OS94 (NCT00180908)	Adriamycin; methotrexate	AM	Methotrexate; ifosfamide; etoposide	MIE
Paul A. Meyers [32]	1998	MSKCC (T12) protocol	Adriamycin; bleomycin; cyclophosphamide; dactinomycin; methotrexate; cisplatin	ABCDMP	Bleomycin; cyclophosphamide; dactinomycin; methotrexate;	BCDM
Robert L. Souhami [33]	1997	EOI (T10) protocol	Adriamycin; bleomycin; cyclophosphamide; dactinomycin; methotrexate; vincristine	ABCDMPL	Adriamycin; cisplatin	AP
Michael P. Link [34]	1993	MIOS	Adriamycin; bleomycin; cyclophosphamide; dactinomycin; methotrexate; cisplatin	ABCDMP	Blank	Blank
John H. Edmonson [35]	1984	Mayo Clinic	Methotrexate; vincristine	ML	Blank	Blank
K. Winkler [36]	1984	COSS-80	Adriamycin; bleomycin; cyclophosphamide; dactinomycin; methotrexate; interferon	ABCDMF	Adriamycin; methotrexate; cisplatin; interferon	AMPF
			Adriamycin; bleomycin; cyclophosphamide; dactinomycin; methotrexate;	ABCDM	Adriamycin; methotrexate; cisplatin	AMP
F. Eilber [37]	1987	NA	Adriamycin; bleomycin; cyclophosphamide; dactinomycin; methotrexate;	ABCDM	Blank	Blank
D.R. Sweetnam [38]	1986	NA	Adriamycin; methotrexate; vincristine	AML	Methotrexate; vincristine	ML
K. Winkler [39]	1988	COSS-82	Adriamycin; bleomycin; cyclophosphamide; dactinomycin; methotrexate; cisplatin; ifosfamide	ABCDMPI	Adriamycin; bleomycin; cyclophosphamide; dactinomycin; methotrexate; cisplatin;	ABCDMP
Vivien H.C. Bramwell [40]	1992	EOI	Adriamycin; methotrexate; cisplatin	AMP	Adriamycin; cisplatin	AP
John C. Ivins [41]	1976	Mayo Clinic	Transfer factor	N	Adriamycin; methotrexate; vincristine	AML
C. Jasmin [42]	1978	EORTC	Adriamycin; cyclophosphamide; methotrexate; vincristine	ACML	Adriamycin; methotrexate; cyclophosphamide; alkeran	ACMK
Gilchrist GS [43]	1978	NA	Adriamycin; methotrexate; vincristine	AML	Transfer factor	N
J.M.V. Burgers [44]	1988	EORTC-SIOP03 (20781)	Adriamycin; cyclophosphamide; methotrexate; vincristine	ACML	Blank	Blank

*Abbreviations*: *CCG* Children’s Cancer Group, *COSS* Cooperative Osteosarcoma Study Group, *EOI* the European Osteosarcoma Intergroup, *EORTC* European Organization for Research on Treatment of Cancer, *EURAMOS-1* The European and American Osteosarcoma Study Group, *MIOS* The Multi-institutional Osteosarcoma Study, *MSKCC* Memorial Sloan Kettering Cancer Center, *SFOP* Societe Francaise d’Oncologie Pediatrique, *SSG* the Scandinavian Sarcoma Group, *NA* not available

**Fig. 2:**
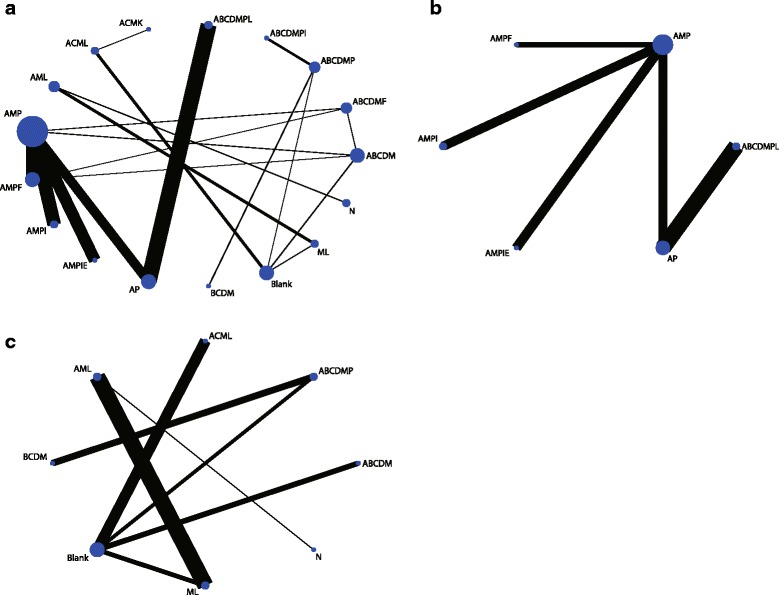
Network of comparisons for all outcomes included in the analyses. **a** Progression-free survival. **b** Overall survival, part one. **c** Overall survival, part two. *Abbreviations*: *A* adriamycin, *B* bleomycin, *C* cyclophosphamide, *D* dactinomycin, *E* etoposide, *F* interferon, *I* ifosfamide, *K* alkeran, *L* vincristine, *M* methotrexate, *N* transfer factor, *P* cisplatin

**Fig. 3:**
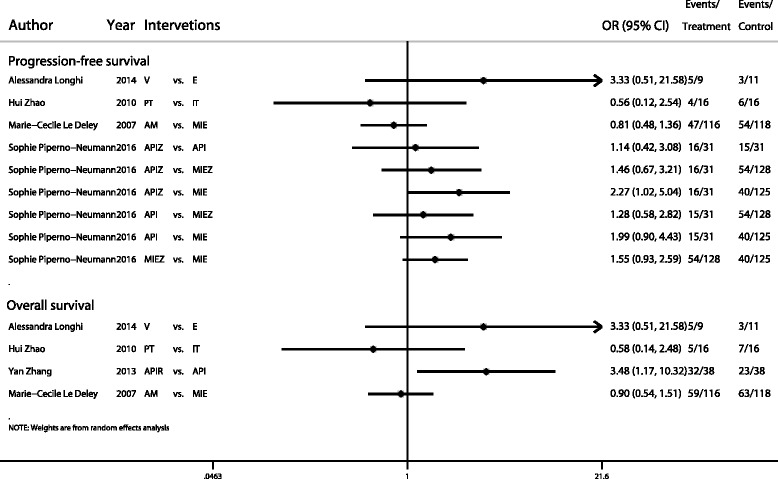
Forest plot of comparisons not included in the network meta-analysis. *Abbreviations*: *A* adriamycin, *E* etoposide, *I* ifosfamide, *M* methotrexate, *P* cisplatin, *R* recombinant human endostatin, *T* pirarubicin, *V Viscum album*, *Z* zoledronate

For the PFS analysis, we extracted all studies of 5-year PFS or the longest follow-up period for PFS. In the present study, we analysed 16 types of multi-drug regimens. Four multi-drug regimens were directly compared to a blank control, which indicated treatment without chemotherapy. In this analysis, the nodes were weighted according to the number of studies evaluated for each treatment, and the edges were weighted according to the precision of the direct estimate for each pairwise comparison (Fig. 2a). In network pairwise comparisons, the ABCDM (all protocol abbreviations are defined in Table 2) regimen was superior to the ACML (logOR, 1.38; 95% CI, 0.09–2.68) and Blank (logOR, 1.30; 95% CI, 0.19–2.41) regimens for the PFS outcome. The ABCDMP regimen was superior to the ACML (logOR, 2.14; 95% CI, 0.45–3.84), AML (logOR, 2.13; 95% CI, 0.00–4.26), Blank (logOR, 2.06; 95% CI, 0.50–3.62), and ML regimens (logOR, 2.24; 95% CI, 0.22–4.27), and the ABCDMPI regimen, combining ABCDMP with ifosfamide, was superior to the ABCDMP regimen alone (logOR, 0.84; 95% CI, 0.09–1.59). The ABCDMPI regimen was also superior to the ACML (logOR, 2.98; 95% CI, 1.13–4.84), AML (logOR, 2.97; 95% CI, 0.71–5.23), Blank (logOR, 2.90; 95% CI, 1.17–4.63), ML (logOR, 3.08; 95% CI, 0.92–5.24), and N (logOR, 2.75; 95% CI, 0.22–5.28) regimens in the network comparisons. Moreover, the ABCDMP regimen in combination with vincristine (ABCDMPL) was superior to the ACML (logOR, 1.89; 95% CI, 0.14–3.64), AMP (logOR, 0.46; 95% CI, 0.01–0.90), AMPF (logOR, 0.64; 95% CI, 0.11–1.18), and Blank (logOR, 1.80; 95% CI, 0.19–3.42) regimens. No other significant differences were found among these regimens (Additional file 2: Table S1). Based on the SUCRA rank, the ABCDMPI regimen was the most likely treatment to improve PFS in osteosarcoma patients (94.1%), followed by the ABCDMPL (81.9%) and ABCDMP (76.9%) regimens. Additionally, the comparison-adjusted funnel plot used to assess publication bias and determine the presence of small-study effects did not suggest any publication bias (Additional file 3: Figure S2a). In addition, some regimens were not included in the network meta-analysis, reflecting a disconnection, and a traditional meta-analysis showed no significant difference between interventions, except for APIZ compared to MIE (OR, 2.27; 95% CI 1.02–5.04) (Fig. 3).

For the OS analysis, we separated the regimens into two groups, reflecting the disconnection. The first group included AMP, AMPF, AMPI, AMPIE, AP, and ABCDMPL. Four regimens were directly compared to AMP, and we directly compared AP and ABCDMPL (Fig. 2b). In the network comparisons, the ABCDMPL regimen showed a significant advantage compared to the AMP (logOR, 0.47; 95% CI, 0.02–0.92), AMPF (logOR, 0.65; 95% CI, 0.01–1.29), and AP (logOR, 0.31; 95% CI, 0.04–0.57) regimens (Additional file 4: Table S2). The results showed that ABCDMPL was most likely the best regimen for improving the OS (94.7%) of osteosarcoma patients, followed by the AP (58.3%) and AMPIE (56.8%) regimens. The second group included ABCDM, ABCDMP, ACML, BCDM, ML, and N. Four regimens were directly compared to the Blank condition (Fig. 2c). In the network comparison, the ABCDM regimen was superior to the AML (logOR, 1.99; 95% CI, 0.14–3.84), Blank (logOR, 1.54; 95% CI, 0.37–2.70), and ML (logOR, 1.76; 95% CI, 0.03–3.49) regimens, and no other significant difference was found among comparisons (Additional file 5: Table S3). Regarding rank, ABCDM (89.4%) was most likely to be the best regimen, followed by N (70.1%) and BCDM (60.9%). The comparison-adjusted funnel plot showed no obvious publication bias (Additional file 3: Figure S2b and c). A comparison of regimens not included in the network meta-analysis revealed that APIR had a significant advantage over API in improving the OS (OR, 3.48; 95% CI, 1.17–10.32) of the patients (Fig. 3). However, this result was based on a single study and lacked precision and robustness.

## Discussion

In the present study, we analysed single- or multi-drug regimens of chemotherapy for the treatment of osteosarcoma using a network meta-analysis. We did not analyse the chemotherapeutic effect according to protocols because the application stage, duration, and dosage of each drug varied. The PFS analysis showed that the ABCDMPI, ABCDMPL, and ABCDMP regimens were most likely to improve PFS in osteosarcoma patients. In the present study, the ABCDMP regimen played a critical role in a treatment involving the T12 protocol (including adriamycin, bleomycin, cyclophosphamide, dactinomycin, methotrexate, cisplatin) used at the Memorial Sloan Kettering Cancer Center (MSKCC) between 1986 and 1993. This more intensive preoperative regimen comprised two courses of cisplatinum and doxorubicin in addition to a high dose of methotrexate and bleomycin, cyclophosphamide, and dactinomycin [32]; it showed a better effect on prolonging the PFS of patients when combined with ifosfamide or vincristine. However, these results are partially supported by a previous view that ifosfamide-based chemotherapy significantly improves the PFS of osteosarcoma patients [7]. In the secondary outcome analysis, we also observed that the regimens with more types of drugs showed better results, but use of a transfer factor also showed advantages. However, these results should be considered with caution, as most studies changed the initial protocol and required more active chemotherapy with metastasis or progression. Therefore, the effective gap between interventions could be reduced, resulting in bias. The practice of changing the chemotherapy regimen is common, correct, and ethical in clinical practice.

Despite the present results, it is undeniable that when the number of different types of chemotherapeutic drugs increases, the cytotoxicity and adverse effects will also simultaneously increase. Thus, a balance exists, suggesting that multi-drug regimens could significantly prolong the PFS of osteosarcoma patients but lead to more serious adverse effects. Adverse effects are common in chemotherapy and include nephrotoxicity, ototoxicity, and bone marrow suppression. Serious adverse effects will affect the application of the chemotherapy programme and even the quality of life of patients.

Thus, in clinical practice, cytoprotective agents, such as muramyl tripeptide, are also frequently and simultaneously used for chemotherapy. However, this agent is not widely used, and the literature did not show that cytoprotective agents significantly improved the PFS and OS of patients [29]. Therefore, in the present study, we did not analyse the use of cytoprotective agents. In addition, *Viscum album*, transfer factor, and recombinant human endostatin are non-traditional chemotherapy drugs that show a cytotoxicity effect. Although they are controversial, we still included these types of drugs in the present analysis.

In the present study, neoadjuvant chemotherapy was used in most included studies. Neoadjuvant chemotherapy includes the administration of chemotherapeutic agents prior to the main treatment, and this regimen has several advantages: (1) It can eliminate micrometastases early to avoid metastases caused by delayed surgery or low resistance. (2) It can control the primary tumour and reduce the chance of surgical tumour spread. (3) It can assess the chemotherapeutic effect and guide the postoperative chemotherapy. (4) It can assess the prognosis earlier. Although the results of RCTs suggested no significant effect on the outcome of patients when comparing preoperative chemotherapy to postoperative chemotherapy [45], neoadjuvant chemotherapy for limb salvage and the surgical process is still worthy of clinical application.

In addition, several studies compared intra-arterial or intravenous chemotherapeutic infusion. When the same regimens were applied, no significant differences were observed in the chemotherapy response between intra-arterial and intravenous infusion [46, 47]. However, some studies suggested that intra-arterial infusion has a more active effect [48, 49]. Regarding the dosage of chemotherapeutic agents, comparisons of a high or moderate dose of methotrexate have primarily been described. A high dose of methotrexate was more widely used in patients who could tolerate this drug. However, in small-sample RCTs of children with osteosarcoma, a significant difference in outcome was not observed between different dosages [50–52].

We systematically analysed chemotherapeutic regimens for osteosarcoma patients using a network meta-analysis, although individual chemotherapeutic protocols could not be analysed. In the present study, multi-drug regimens, such as the T12 protocol plus ifosfamide or vincristine, had a better effect on prolonging the PFS and OS of osteosarcoma patients. Further research with well-designed, double-blinded RCTs is still necessary, as the psychological evidence might also influence patient outcomes. In addition, further trials using relatively well-developed chemotherapeutic protocols would be beneficial to analyse the differences among multiple chemotherapeutic protocols.

### Limitations

There are several limitations to the present study. First, the present analysis was performed at a study level, not at an individual level. Second, for chemotherapy, cytoprotective agents might also improve the survival time of patients by reducing the chemotherapy-induced damage to normal tissue, but these drugs were not analysed in this study. Third, we did not perform the Grading of Recommendations Assessment, Development and Evaluation in the present analysis, as all included studies had an RCT design without blind concealment, and most of the results showed a low risk of imprecision.

## Conclusions

In conclusion, the T12 protocol has a better effect on prolonging the PFS of osteosarcoma patients when combined with ifosfamide or vincristine. For the OS, the T12 protocol plus vincristine or the removal of cisplatinum also represents the best regimen. Further RCTs of chemotherapeutic protocols are still necessary.

## Additional files

Additional file 1: Figure S1. Risk of bias graph of each included study. (EPS 2203 kb)

Additional file 2: Table S1. The league table of the network for the progression-free survival estimates the treatments according to their relative effects. (DOCX 19 kb)

Additional file 3: Figure S2. Comparison-adjusted funnel plots for assessing all outcomes. a. Progression-free survival; b. Overall survival, part one; c. Overall survival, part two. (EPS 888 kb)

Additional file 4: Table S2. The league table of the network for the overall survival estimates the treatments according to their relative effects for first part. (DOCX 14 kb)

Additional file 5: Table S3. The league table of the network for the overall survival estimates the treatments according to their relative effects for second part. (DOCX 14 kb)

## Abbreviations

A: Adriamycin
B: Bleomycin
C: Cyclophosphamide
CCG: Children’s Cancer Group
COSS: Cooperative Osteosarcoma Study Group
CTs: Confidence intervals
D: Dactinomycin
E: Etoposide
EOI: The European Osteosarcoma Intergroup
EORTC: European Organization for Research on Treatment of Cancer
EURAMOS-1: The European and American Osteosarcoma Study Group
F: Interferon
G-CSF: Granulocyte colony stimulating factor
I: Ifosfamide
K: Alkeran
L: Vincristine
M: Methotrexate
MIOS: The Multi-institutional Osteosarcoma Study
MSKCC: Memorial Sloan Kettering Cancer Center
N: Transfer factor
NA: Not available
ORs: Odds ratios
OS: Overall survival
P: Cisplatin
PFS: Progression-free survival
PRISMA: Preferred Reporting Items for Systematic Reviews
R: Recombinant human endostatin
RCT: Randomized controlled trial
SFOP: Societe Francaise d’Oncologie Pediatrique
SSG: The Scandinavian Sarcoma Group
SUCRA: Surface Under the Cumulative Ranking
T: Pirarubicin
V: *Viscum album*
Z: Zoledronate
