# Supplementary material for: Effectiveness of multi-drug regimen chemotherapy treatment in osteosarcoma patients: a network meta-analysis of randomized controlled trials
Source: J Orthop Surg Res. 2017 Mar 29;12:52. doi: 10.1186/s13018-017-0544-9 (PMC5372345; doi:10.1186/s13018-017-0544-9)
Supplement: Supplementary file 2 — The league table of the network for the progression-free survival estimates the treatments according to their relative effects. (DOCX 19 kb) [file 13018_2017_544_MOESM2_ESM.docx]

Additional file 2: Table S1. The league table of the network for the progression-free survival estimates the treatments according to their relative effects.

| **ABCDM** |  |  |  |  |  |  |  |  |  |  |  |  |  |  |  |  |
| --- | --- | --- | --- | --- | --- | --- | --- | --- | --- | --- | --- | --- | --- | --- | --- | --- |
| -0.04 (-1.32,1.25) | **ABCDMF** |  |  |  |  |  |  |  |  |  |  |  |  |  |  |  |
| -0.76 (-2.67,1.15) | -0.72 (-3.03,1.58) | **ABCDMP** |  |  |  |  |  |  |  |  |  |  |  |  |  |  |
| -1.60 (-3.65,0.46) | -1.56 (-3.98,0.86) | **-0.84 (-1.59,-0.09)** | **ABCDMPI** |  |  |  |  |  |  |  |  |  |  |  |  |  |
| -0.50 (-1.68,0.67) | -0.47 (-1.75,0.82) | 0.26 (-1.99,2.50) | 1.09 (-1.28,3.46) | **ABCDMPL** |  |  |  |  |  |  |  |  |  |  |  |  |
| 0.63 (-1.39,2.64) | 0.66 (-1.72,3.05) | 1.39 (-0.90,3.68) | 2.22 (-0.19,4.64) | 1.13 (-1.20,3.46) | **ACMK** |  |  |  |  |  |  |  |  |  |  |  |
| **1.38 (0.09,2.68)** | 1.42 (-0.40,3.24) | **2.14 (0.45,3.84)** | **2.98 (1.13,4.84)** | **1.89 (0.14,3.64)** | 0.76 (-0.78,2.30) | **ACML** |  |  |  |  |  |  |  |  |  |  |
| 1.37 (-0.46,3.20) | 1.41 (-0.83,3.64) | **2.13 (0.00,4.26)** | **2.97 (0.71,5.23)** | 1.87 (-0.30,4.05) | 0.74 (-1.48,2.96) | -0.01 (-1.61,1.59) | **AML** |  |  |  |  |  |  |  |  |  |
| -0.05 (-1.14,1.04) | -0.01 (-1.22,1.20) | 0.71 (-1.49,2.91) | 1.55 (-0.78,3.88) | **0.46 (0.01,0.90)** | -0.67 (-2.96,1.61) | -1.43 (-3.12,0.26) | -1.42 (-3.55,0.71) | **AMP** |  |  |  |  |  |  |  |  |
| 0.14 (-0.96,1.25) | 0.18 (-1.04,1.40) | 0.90 (-1.31,3.11) | 1.74 (-0.59,4.07) | **0.64 (0.11,1.18)** | -0.49 (-2.78,1.81) | -1.24 (-2.95,0.46) | -1.23 (-3.36,0.90) | 0.19 (-0.11,0.49) | **AMPF** |  |  |  |  |  |  |  |
| -0.02 (-1.14,1.11) | 0.02 (-1.22,1.26) | 0.75 (-1.48,2.97) | 1.58 (-0.76,3.93) | 0.49 (-0.05,1.02) | -0.64 (-2.95,1.66) | -1.40 (-3.12,0.32) | -1.39 (-3.53,0.76) | 0.03 (-0.26,0.33) | -0.16 (-0.58,0.26) | **AMPI** |  |  |  |  |  |  |
| -0.07 (-1.21,1.06) | -0.04 (-1.29,1.21) | 0.69 (-1.54,2.91) | 1.52 (-0.82,3.87) | 0.43 (-0.12,0.98) | -0.70 (-3.01,1.61) | -1.46 (-3.18,0.26) | -1.44 (-3.59,0.71) | -0.03 (-0.34,0.29) | -0.21 (-0.65,0.22) | -0.06 (-0.49,0.37) | **AMPIE** |  |  |  |  |  |
| -0.24 (-1.39,0.91) | -0.20 (-1.46,1.06) | 0.52 (-1.71,2.75) | 1.36 (-1.00,3.71) | 0.26 (-0.01,0.53) | -0.87 (-3.18,1.45) | -1.62 (-3.35,0.11) | -1.61 (-3.77,0.55) | -0.19 (-0.55,0.16) | -0.38 (-0.85,0.08) | -0.22 (-0.69,0.24) | -0.17 (-0.64,0.31) | **AP** |  |  |  |  |
| -0.50 (-2.69,1.69) | -0.46 (-3.01,2.08) | 0.26 (-0.81,1.33) | 1.10 (-0.21,2.40) | 0.00 (-2.49,2.49) | -1.13 (-3.66,1.40) | -1.89 (-3.89,0.12) | -1.87 (-4.26,0.51) | -0.45 (-2.90,1.99) | -0.64 (-3.10,1.81) | -0.49 (-2.95,1.98) | -0.43 (-2.90,2.04) | -0.26 (-2.73,2.21) | **BCDM** |  |  |  |
| **1.30 (0.19,2.41)** | 1.34 (-0.36,3.03) | **2.06 (0.50,3.62)** | **2.90 (1.17,4.63)** | **1.80 (0.19,3.42)** | 0.67 (-1.00,2.35) | -0.08 (-0.75,0.59) | -0.07 (-1.52,1.38) | 1.35 (-0.21,2.90) | 1.16 (-0.41,2.72) | 1.32 (-0.27,2.90) | 1.37 (-0.21,2.96) | 1.54 (-0.05,3.14) | 1.80 (-0.09,3.69) | **Blank** |  |  |
| 1.48 (-0.22,3.18) | 1.52 (-0.61,3.65) | **2.24 (0.22,4.27)** | **3.08 (0.92,5.24)** | 1.99 (-0.08,4.05) | 0.86 (-1.26,2.97) | 0.10 (-1.36,1.55) | 0.11 (-0.56,0.78) | 1.53 (-0.49,3.55) | 1.34 (-0.69,3.37) | 1.50 (-0.54,3.54) | 1.56 (-0.49,3.60) | 1.72 (-0.33,3.77) | 1.98 (-0.31,4.27) | 0.18 (-1.11,1.47) | **ML** |  |
| 1.15 (-1.00,3.31) | 1.19 (-1.32,3.70) | 1.91 (-0.51,4.33) | **2.75 (0.22,5.28)** | 1.66 (-0.80,4.11) | 0.53 (-1.97,3.03) | -0.23 (-2.20,1.74) | -0.22 (-1.36,0.93) | 1.20 (-1.21,3.62) | 1.01 (-1.41,3.44) | 1.17 (-1.26,3.60) | 1.23 (-1.21,3.66) | 1.39 (-1.05,3.84) | 1.65 (-0.99,4.30) | -0.15 (-2.00,1.70) | -0.33 (-1.66,1.00) | **N** |
