# Supplementary material for: Effectiveness of multi-drug regimen chemotherapy treatment in osteosarcoma patients: a network meta-analysis of randomized controlled trials
Source: J Orthop Surg Res. 2017 Mar 29;12:52. doi: 10.1186/s13018-017-0544-9 (PMC5372345; doi:10.1186/s13018-017-0544-9)
Supplement: Supplementary file 4 — The league table of the network for the overall survival estimates the treatments according to their relative effects for first part. (DOCX 14 kb) [file 13018_2017_544_MOESM4_ESM.docx]

Additional file 4: Table S2. The league table of the network for the overall survival estimates the treatments according to their relative effects for first part.

| **ABCDMPL** |  |  |  |  |  | |
| --- | --- | --- | --- | --- | --- | --- |
| **0.47 (0.02,0.92)** | **AMP** |  |  |  |  | |
| **0.65 (0.01,1.29)** | 0.18 (-0.27,0.64) | **AMPF** |  |  |  | |
| 0.44 (-0.18,1.05) | -0.03 (-0.41,0.35) | -0.21 (-0.80,0.38) | **AMPI** |  |  | |
| 0.34 (-0.22,0.91) | -0.13 (-0.47,0.21) | -0.31 (-0.88,0.26) | -0.09 (-0.60,0.42) | **AMPIE** |  | |
| **0.31 (0.04,0.57)** | -0.16 (-0.54,0.22) | -0.35 (-0.94,0.25) | -0.13 (-0.70,0.44) | -0.04 (-0.55,0.47) | **AP** |  |
