# Supplementary material for: Effectiveness of multi-drug regimen chemotherapy treatment in osteosarcoma patients: a network meta-analysis of randomized controlled trials
Source: J Orthop Surg Res. 2017 Mar 29;12:52. doi: 10.1186/s13018-017-0544-9 (PMC5372345; doi:10.1186/s13018-017-0544-9)
Supplement: Supplementary file 5 — The league table of the network for the overall survival estimates the treatments according to their relative effects for second part. (DOCX 14 kb) [file 13018_2017_544_MOESM5_ESM.docx]

Additional file 5: Table S3. The league table of the network for the overall survival estimates the treatments according to their relative effects for second part.

| **ABCDM** |  |  |  |  |  |  |  |
| --- | --- | --- | --- | --- | --- | --- | --- |
| 1.54 (-0.33,3.41) | **ABCDMP** |  |  |  |  |  |  |
| 1.18 (-0.23,2.59) | -0.36 (-2.02,1.30) | **ACML** |  |  |  |  |  |
| **1.99 (0.14,3.84)** | 0.45 (-1.60,2.50) | 0.81 (-0.84,2.46) | **AML** |  |  |  |  |
| 0.98 (-1.16,3.12) | -0.56 (-1.61,0.49) | -0.20 (-2.17,1.77) | -1.01 (-3.31,1.29) | **BCDM** |  |  |  |
| **1.54 (0.37,2.70)** | 0.00 (-1.46,1.46) | 0.36 (-0.44,1.16) | -0.45 (-1.89,0.99) | 0.56 (-1.24,2.36) | **Blank** |  |  |
| **1.76 (0.03,3.49)** | 0.22 (-1.72,2.16) | 0.58 (-0.93,2.09) | -0.23 (-0.89,0.43) | 0.78 (-1.42,2.99) | 0.22 (-1.05,1.50) | **ML** |  |
| 0.33 (-3.23,3.89) | -1.21 (-4.88,2.45) | -0.85 (-4.31,2.61) | -1.66 (-4.71,1.38) | -0.65 (-4.47,3.16) | -1.21 (-4.58,2.15) | -1.44 (-4.55,1.68) | **N** |
